# A sex-sensitive LC-MS/MS method with isotopic internal standard for prazosin bioequivalence: bridging precision medicine and generic drug policy in China

**DOI:** 10.3389/fphar.2025.1592731

**Published:** 2025-05-23

**Authors:** Hui Chen, Junping Guo, Zhenyu Zhu, Xiaomei Huang, Jincai Guo

**Affiliations:** ^1^ Department of Pharmacy, Changsha Stomatological Hospital, Changsha, China; ^2^ School of Stomatology, Hunan University of Chinese Medicine, Changsha, China; ^3^ Department of Pharmacy, Hubei Province Corps Hospital, The Chinese Armed Police Force (CAPF), Wuhan, China; ^4^ Department of Phase I Clinical Trial Research Center, XiangYa BoAi Rehabilitation Hospital, Changsha, China

**Keywords:** prazosin, prazosin-d8, LC-MS/MS, pharmacokinetics, bioequivalence

## Abstract

**Objective:**

To develop a rapid, sensitive, and high-throughput liquid chromatography-tandem mass spectrometry (LC-MS/MS) method for prazosin quantification in human plasma, validate its application in bioequivalence studies, and investigate sex-specific pharmacokinetic differences in a Chinese population.

**Materials and methods:**

Plasma samples were processed by protein precipitation with methanol and analyzed using a Waters ACQUITY UPLC^®^ HSS T3 column. Prazosin-d8 was used as an isotopic internal standard (IS) to enhance quantification accuracy. Chromatographic separation was performed with methanol (A) and 0.1% formic acid in water (B) as the mobile phases, using gradient elution at 0.35 mL/min. Quantification was achieved using positive ionization mode with multiple reaction monitoring (MRM) transitions of m/z 384.2→95.0 for prazosin and m/z 392.2→95.0 for IS.

**Results:**

The method demonstrated excellent linearity (0.1000–30.00 ng/mL, LLOQ: 0.1000 ng/mL), surpassing the sensitivity of prior methods. Bioequivalence analysis confirmed that the 90% confidence interval (CI) for AUC_0-24_, AUC_0-∞_, and C_max_ geometric mean ratios fell within the regulatory acceptance range (90.00%–111.11%). Sex analysis revealed significantly higher AUC_0-24_ (+48%) and AUC_0-∞_ (+46%) medians in females (n = 4) than in males (n = 16) (*P* < 0.05), suggesting potential sex-based differences in prazosin pharmacokinetics.

**Conclusion:**

This study establishes the first LC-MS/MS method integrating isotopic IS and sex-specific pharmacokinetic profiling for prazosin, offering regulatory-compliant bioequivalence validation and insights into precision dosing strategies. These findings support China’s generic drug policy and highlight the need for sex-stratified pharmacokinetic evaluations in bioequivalence assessments.

**Clinical trial registration number:**

ChiCTR2100050626.

## 1 Introduction

Prazosin, a selective α1-adrenergic receptor antagonist, has been widely used to treat hypertension since 1976 (Stanaszek et al., 1983). More recently, its therapeutic indications have expanded to neuropsychiatric disorders, including post-traumatic stress disorder (PTSD) and alcohol use disorder (AUD), owing to its ability to cross the blood-brain barrier and regulate stress responses (Hendrickson and Raskind, 2016; Ferrafiat et al., 2020; Eichelman and Dorava, 2021; Reist et al., 2021; Karaoglan and Grace, 2025). Clinical trials suggest that prazosin alleviates PTSD-related nightmares and reduces AUD relapse rates by attenuating noradrenergic hyperactivity (Gilpin and Weiner, 2017; Kleinman and Ostacher, 2019). However, despite its widespread use, pharmacokinetic profiling remains insufficiently characterized, particularly in diverse populations, such as the Chinese population, limiting its optimized clinical application and regulatory assessment.

Ensuring the therapeutic equivalence of generic drugs is a critical component of China’s Generic Drug Consistency Evaluation Policy, which mandates bioequivalence studies to bridge the gap between innovators and generic formulations (The central People’s government of the People’s Republic of China, 2025). However, current bioequivalence assessments rarely account for sex-specific pharmacokinetic variability despite increasing evidence that biological sex influences drug absorption, metabolism, and efficacy. The lack of sex-informed regulatory standards may contribute to suboptimal therapeutic outcomes and unintended adverse effects, highlighting a policy gap in precision medicine. Addressing these issues is essential for optimizing drug-dosing strategies and improving clinical outcomes.

Analytical techniques such as high-performance liquid chromatography (HPLC) (Twomey and Hobbs, 1978; Yee et al., 1979; Rathinavelu and Malave, 1995; Andros et al., 1996), fluorescence detection (Chau et al., 1980; Guo et al., 2016), and liquid chromatography-mass spectrometry (LC-MS) (Erve et al., 2008) have been used for prazosin quantification in plasma. However, HPLC often lacks sensitivity and throughput, and fluorescence-based methods are expensive and impractical for routine drug monitoring. LC-MS offers high selectivity and sensitivity (Seger and Salzmann, 2020; Loh et al., 2022; Maekawa and Mano, 2022); however, existing methods lack the robustness required for large-scale pharmacokinetic and bioequivalence studies across diverse populations, particularly in regulatory settings. Additionally, conventional sample preparation techniques, such as liquid-liquid extraction and solid-phase extraction, are labor-intensive and costly (Gurule et al., 2010; Liu et al., 2021), limiting their applicability for regulatory bioequivalence testing. Simpler alternatives, such as protein precipitation, have been explored, but remain inadequately validated for human plasma samples, creating a barrier to regulatory approval and policy adoption.

This study aimed to develop a sex-sensitive, high-throughput LC-MS/MS method to generate critical evidence for policy discussions on sex-inclusive pharmacokinetic evaluations, personalized dosing guidelines, and regulatory standards for generic drug bioequivalence in China. This study contributes to pharmaceutical policy refinement and advances in precision medicine by bridging bioanalytical innovations with real-world regulatory and clinical applications.

## 2 Materials and Methods

### 2.1 Development and validation of the LC-MS/MS method

To support this bioequivalence study, a high-throughput and sensitive LC-MS/MS method was developed for the quantification of prazosin in human plasma. This method was designed to ensure accuracy, precision, specificity, and regulatory compliance, particularly for detecting sub-therapeutic concentrations at low plasma levels. The analysis was performed using an ultra-performance liquid chromatography-tandem mass spectrometry (UPLC-MS/MS) system consisting of an ACQUITY UPLC I-CLASS system (Waters, United States) coupled to a triple quadrupole mass spectrometer (AB Sciex Triple Quad 5500, AB Sciex, United States). Chromatographic separation was conducted using an ACQUITY UPLC HSS T3 column (1.8 μm, 2.1 × 50 mm, Waters, United States), maintained at 40°C, with a flow rate of 0.35 mL/min. The mobile phase consisted of methanol (A) and 0.1% formic acid in water (B), using a gradient elution program: 0–0.5 min: 35% A; 0.5–1.8 min: 35% → 98% A; 1.8–2.8 min: 98% A; and 2.8–3.5 min: 98% → 35% A. The autosampler was maintained at 6°C and the injection volume was 4.0 μL.

Detection was performed in the positive ionization mode using multiple reaction monitoring (MRM) with optimized transitions for prazosin (m/z 384.2 → 95.0) and prazosin-d8 (internal standard, IS) (m/z 392.2 → 95.0). Calibration curves were prepared in blank plasma using prazosin standard solutions ranging from 0.1000 to 30.00 ng/mL. The lower limit of quantification (LLOQ) was defined as the lowest analyte concentration measurable with accuracy (±20%) and precision (coefficient of variation (CV) ≤ 20%). The lowest calibration standard (0.1000 ng/mL) was validated as the LLOQ to simplify method implementation while maintaining sensitivity.

Plasma samples (50 µL) were spiked with 10 µL prazosin-d8 (IS) working solution (10 ng/mL), followed by protein precipitation using 300 µL methanol. The mixture was vortexed for 8 min, centrifuged at 5500 × g for 10 min, and the supernatant (100 µL) was transferred and diluted with 200 µL of ultrapure water. A final 4 µL sample was injected into the LC-MS/MS system.

### 2.2 Bioequivalence study design

This single-center, randomized, open-label, two-period, single-dose crossover bioequivalence trial was conducted under fasting conditions. The trial followed the China NMPA bioequivalence guidelines, adhered to Good Clinical Practice (GCP) and the Declaration of Helsinki, and was approved by the Medical Ethics Committee of Xiangya Boai Rehabilitation Hospital, Hunan Province, China (Clinical Trial Registration Number: ChiCTR2100050626). The trial was performed between 2021.11.01 and 2021.12.21, with written informed consent obtained from all participants.

The sample size was determined in accordance with the China NMPA bioequivalence guidelines (National Medical Products Administration, 2016; National Medical Products Administration, 2020), which mandate a minimum of 18 subjects. To ensure statistical robustness and account for potential attrition, 20 healthy subjects (16 males and 4 females) were enrolled, meeting the following criteria: age ≥18 years, body mass index (BMI) between 19 and 26 kg/m^2^, male weight ≥50 kg, and female weight ≥45 kg. Volunteers with medical conditions affecting drug metabolism (nervous, digestive, and circulatory systems), drug sensitivity, or medication use within 30 days prior to the study were excluded. Each subject underwent comprehensive medical screening, including physical examination, laboratory tests, ECG, and vital sign assessments.

The study followed a biperiodic crossover design, in which each participant received both the test formulation (prazosin 2 mg tablet, Lot: G139210806, APT PHARMA LIMITED) and the reference formulation (Minipres^®^ 2 mg tablet, Lot: M01, Farmasierra Manufacturing, S.L., Spain), with a 7-day washout period between doses. All participants fasted for ≥10 h before drug administration. Subjects were randomized to receive either the test or reference formulation first, with water restricted to 1 h before and 2 h after dosing. Standardized meals were provided 4 h and 10 h after administration to minimize dietary effects.

### 2.3 Blood sample collection and processing

Venous blood samples (4 mL each) were collected pre-dose (0 h) and at multiple post-dose time points, including 0.25, 0.5, 1, 1.5, 2, 2.5, 3, 3.5, 4, 5, 6, 7, 8, 10, 12, and 24 h. Following the 7-day washout period, the subjects received the alternate formulation, and identical blood collection procedures were followed. Plasma samples were separated by centrifugation at 5500 × g for 10 min and stored at −80°C before LC-MS/MS analysis.

### 2.4 Pharmacokinetic and sex-specific bioequivalence assessment

Plasma prazosin concentrations were analyzed using Phoenix WinNonlin software (Version 8.3.1). Key pharmacokinetic parameters, including C_max_, AUC_0-24_, and AUC_0-∞_, were calculated for each subject. Bioequivalence was assessed according to the China NMPA criteria, with confirmation achieved if the 90% confidence interval (CI) for the geometric mean ratios (GMR) of C_max_ and AUC fell within the 90.00%–111.11% range.

To evaluate potential sex-based pharmacokinetic variability, a sex-stratified bioequivalence analysis was conducted. Plasma concentration-time data were analyzed separately for male and female participants, and additional pharmacokinetic parameters, including T_max_ and t_1/2_, were compared between sexes. The same LC-MS/MS analytical method and pharmacokinetic evaluation procedures were applied across both groups to ensure consistency in data interpretation. Bioequivalence assessments were performed using Phoenix WinNonlin software (Version 8.3.1), with statistical comparisons between sexes conducted using analysis of variance (ANOVA) and nonparametric tests for T_max_. The 90% CI for the GMR of C_max_ and AUC were calculated separately for the male and female subgroups to determine the potential pharmacokinetic differences.

### 2.5 Statistical analysis

Pharmacokinetic parameters were analyzed using Phoenix WinNonlin software (version 8.3.1), while additional statistical analyses were conducted using SPSS (version 24). AUC and C_max_ were log-transformed and analyzed using ANOVA to assess the variability between formulations. Normality of pharmacokinetic parameters was assessed using the Shapiro–Wilk test (α = 0.05). Non-normal parameters were analyzed using non-parametric methods and reported as median (range). Two one-sided t-tests and 90% CI were used to determine bioequivalence. Sex-based comparisons (Male: n = 16; Female: n = 4) were analyzed using Mann–Whitney U tests, regardless of normality, to ensure conservative interpretation for the small female cohort. Results are reported with 90% CI.

## 3 Results

### 3.1 Method development and validation

The validated LC-MS/MS method demonstrated high sensitivity, specificity, and efficiency, allowing for precise quantification of prazosin in human plasma. The method achieved a LLOQ of 0.1000 ng/mL with a total analysis time of 3.5 min, making it ideal for large-scale pharmacokinetic and bioequivalence studies.

#### 3.1.1 Specificity, linearity, and sensitivity

**FIGURE 1:**
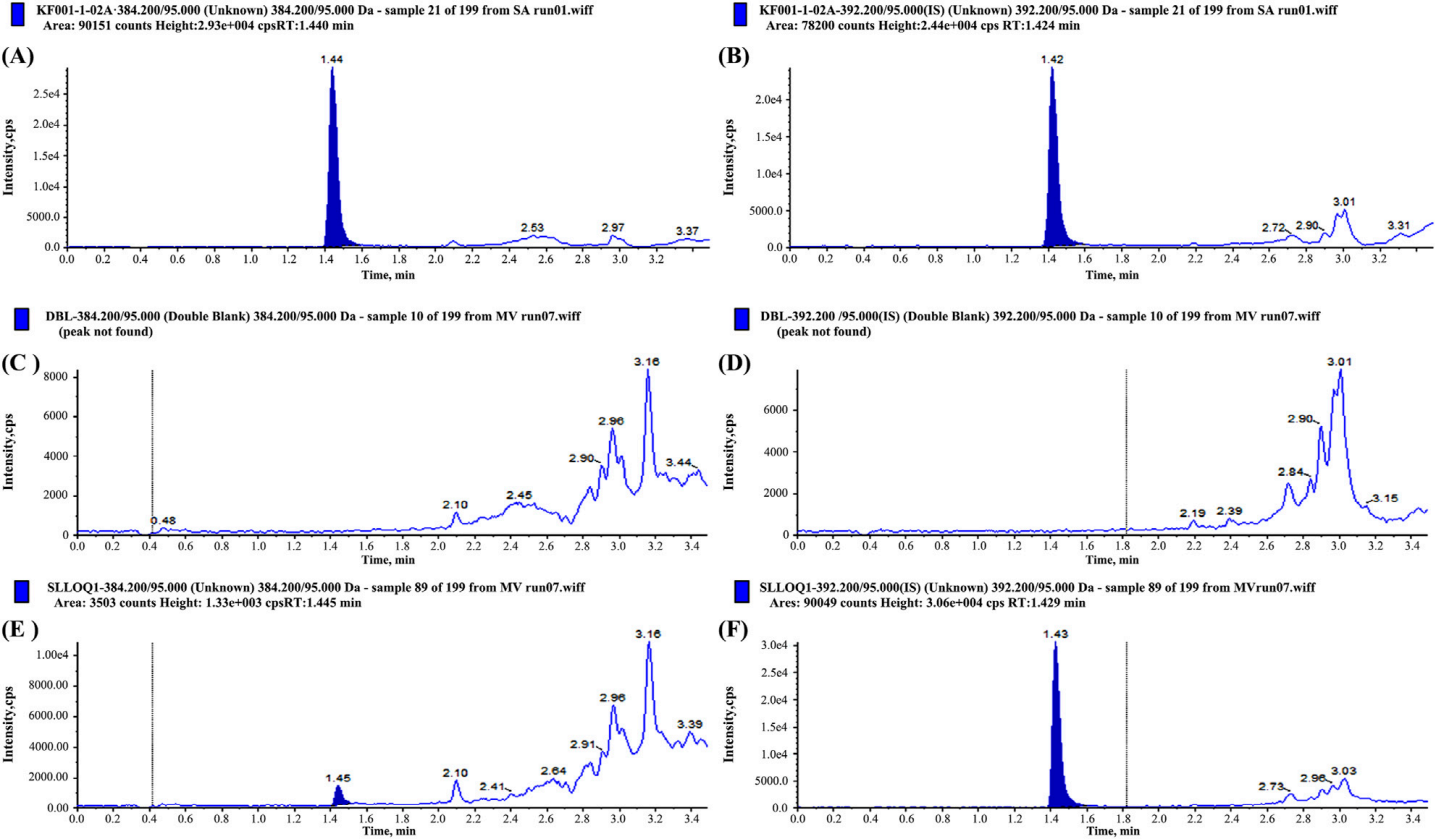
Chromatograms of prazosin and internal standard (IS) in blank plasma **(A,B)**, Lower Limit of Quantitation (LLOQ) sample **(C,D)**, and plasma samples from subjects after administration **(E,F)**, respectively.

The method exhibited high specificity, as confirmed by the absence of significant interference from endogenous plasma components in the blank samples (Figure 1). The calibration curve demonstrated excellent linearity over the validated concentration range (0.1000–30.00 ng/mL), with a correlation coefficient (r) of 0.9989, ensuring accurate quantification across therapeutic and subtherapeutic plasma levels (Table 1).

**TABLE 1:** The calibration curve standards data for prazosin (n = 6).

CC standards	CONC.1	CONC.2	CONC.3	CONC.4	CONC.5	CONC.6	CONC.7	CONC.8	Slope	Intercept	r
Nominal Concentration (ng/mL)	0.1000	0.2000	0.5000	1.000	6.000	12.00	24.00	30.00	0.379	−0.0023	0.9989
Return-mean Value	0.1003	0.2021	0.4890	0.9604	5.895	12.35	24.42	30.58			
%CV	3.1	6.1	2.6	5.9	1.8	1.5	2.1	2.5			
%RE	0.3	1.0	−2.2	−4.0	−1.8	2.9	1.8	1.9			

CC standards, Calibration Curve Standards; CONC, concentration; CV, coefficient of variation; RE, relative error.

#### 3.1.2 Matrix effects and extraction recovery

Matrix effects were evaluated using plasma from six different sources, with variation coefficients below 15%, confirming the method’s robustness for biological sample analysis. The extraction recovery ranged from 89.3% to 96.0%, surpassing the regulatory bioanalytical acceptance criteria and demonstrating high reproducibility and efficiency (Table 2).

**TABLE 2:** Extraction recovery for prazosin from plasma samples.

Concentration (ng/mL)	Post-extraction	Pre-extraction	Recovery (%)	%CV
Prazosin (n = 6, Mean ± SD)				
0.3	11513 ± 547	11992 ± 455	96.0	2.6
2.5	90712 ± 4045	96161 ± 876	94.3	4.0
22.5	842966 ± 13384	944091 ± 12692	89.3	6.5
IS (n = 18, Mean ± SD)				
10.0	94588 ± 1499	103375 ± 3877	91.6	4.7

CV, coefficient of variation; IS, internal standard.

#### 3.1.3 Accuracy, precision, and stability

Inter- and intra-batch accuracy and precision assessments confirmed compliance with regulatory acceptance limits, with CV values below 11.9% (Table 3). Stability assessments confirmed sample integrity across different storage conditions, including short-term storage at room temperature (254 h), multiple freeze-thaw cycles (four cycles), and long-term frozen storage (−20°C for 33 days) (Table 4). These findings support the reliability of this method for pharmacokinetic and bioequivalence studies.

**TABLE 3:** Intra-batch and inter-batch of Accuracy and Precision from plasma samples (n = 6).

Batch	Parameter		Nominal QC concentration (ng/mL)			
			LLOQ (0.1000)	LQC (0.3000)	MQC (2.500)	HQC (22.50)
Intra-batch (n = 6)	Run 1	Accuracy (%RE)	7.6	3.5	1.5	4.1
		Precision (%CV)	7.6	4.4	1.4	1.3
	Run 2	Accuracy (%RE)	−12.6	0.3	0.6	−1.0
		Precision (%CV)	7.5	3.6	0.7	0.7
	Run 3	Accuracy (%RE)	7.1	0.2	−0.3	8.5
		Precision (%CV)	7.2	4.7	0.5	0.6
Inter-batch (n = 18)		Accuracy (%RE)	0.7	1.3	0.6	3.9
		Precision (%CV)	11.9	4.3	1.2	4.0

QC, quality control; LLOQ, lower limit of quantitation; LQC, low quality control; MQC, medium quality control; HQC, high quality control; CV, coefficient of variation; RE, relative error.

**TABLE 4:** Results of stability tests of prazosin and IS (n = 6).

Stability test (storage condition and duration)	Accuracy (%RE)	Precision (%CV)
Short-term, room temperature for 254 h		
LQC (0.3000 ng/mL)	2.6	4.4
HQC (22.50 ng/mL)	−0.5	3.9
Long-term, −20°C for 8 days		
LQC (0.3000 ng/mL)	−2.0	3.4
HQC (22.50 ng/mL)	2.2	4.7
Pre-pretreatment, room temperature for 19 h		
LQC (0.3000 ng/mL)	−4.9	2.4
HQC (22.50 ng/mL)	−6.0	0.7
Pre-pretreatment, ice bath for 19 h		
LQC (0.3000 ng/mL)	−5.4	4.2
HQC (22.50 ng/mL)	−5.7	1.2
Freeze and thaw, <−60°C, 4 cycles		
LQC (0.3000 ng/mL)	−6.5	6.0
HQC (22.50 ng/mL)	−3.6	1.9
Freeze and thaw, −20°C, 4 cycles		
LQC (0.3000 ng/mL)	−4.1	4.1
HQC (22.50 ng/mL)	−5.0	1.7
Long term plasma, <−60°C for 21 days		
LQC (0.3000 ng/mL)	7.2	4.1
HQC (22.50 ng/mL)	2.7	3.0
Long term plasma, −20°C for 21 days		
LQC (0.3000 ng/mL)	6.1	12.7
HQC (22.50 ng/mL)	8.3	2.3
Post-pretreatment, 6°C for 79 h		
LQC (0.3000 ng/mL)	2.1	5.3
HQC (22.50 ng/mL)	2.7	2.1
Stock solution stability (analyte), room temperature for 254 h	−2.6	5.7
Stock solution stability (analyte), −20°C for 33 days	5.3	2.5
Stock solution stability (IS), room temperature for 254 h	−0.7	3.3

IS, internal standard; LQC, low quality control; HQC, high quality control; CV, coefficient of variation; RE, relative error.

### 3.2 Bioequivalence study outcomes

#### 3.2.1 Subject characteristics

Twenty healthy Chinese subjects (4 females and 16 males) were enrolled (Table 5).

**TABLE 5:** Basic information of the subjects.

Parameter	Female (n = 4)	Male (n = 16)
Age (years)	19–27	19–46
Height (cm)	147.0–168.5	162.0–185.0
Weight (kg)	55.4–69.0	55.9–74.6
BMI (kg·m^−2^)	24.3–25.9	20.3–26.0

BMI: body mass index.

#### 3.2.2 Plasma concentration-time profile

**FIGURE 2:**
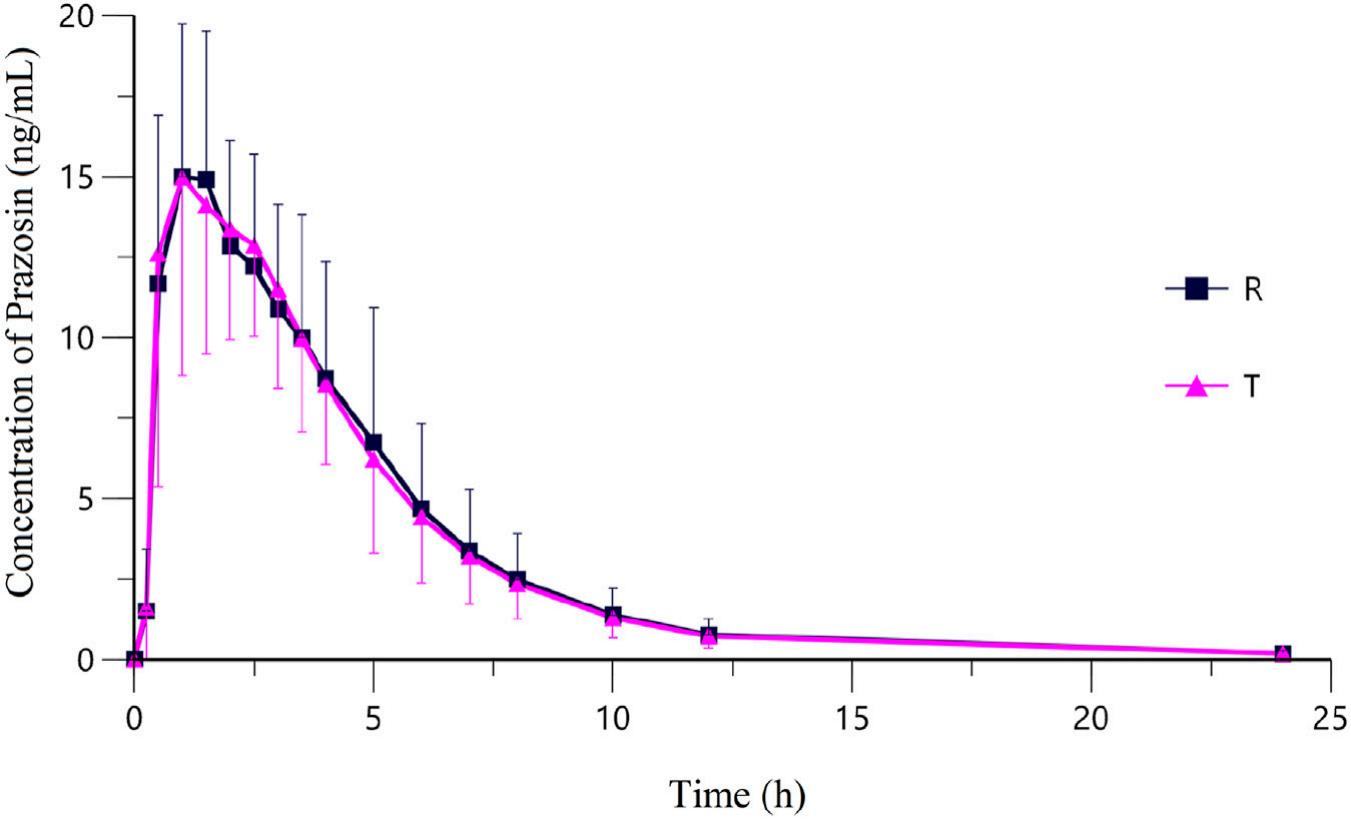
Mean plasma concentration-time curves of Prazosin after a single oral dose of Prazosin Tablets (T: test formulation or R: reference formulation) in 20 subjects under Fasting State. Data from this study support China’s generic drug policy by enabling precise bioequivalence assessment in a representative population.

Following oral administration of 2 mg prazosin under fasting conditions, the validated LC-MS/MS method provided reliable quantification of prazosin concentrations across all sampling time points. The plasma concentration-time curve (Figure 2) revealed a rapid rise in plasma drug levels post-administration, with T_max_ values of 1.00 [0.50-2.50] h for the test formulation and 1.00 [0.50-3.50] h for the reference formulation (Table 6).

**TABLE 6:** Descriptive statistics of main pharmacokinetic parameters (fasting state).

Parameter	Test^a^	Reference^a^	Ratio (T/R, %)	90% CI (lower - upper)
t_1/2_ (h)	4.25 [2.04–10.15]	4.26 [2.09–9.57]		
T_max_ (h)	1.00 [0.50–2.50]	1.00 [0.50–3.50]		
C_max_ (ng/mL)	18.28 ± 4.42	18.17 ± 4.17	100.43	91.57–110.12
AUC_0-24_ (ng·h/mL)	74.95 ± 21.57	75.53 ± 24.84	100.29	94.65–106.26
AUC_0-∞_ (ng·h/mL)	76.66 ± 21.81	77.22 ± 24.97	100.25	94.74–106.07

^a^ Non-normally distributed parameters (t_1/2_ and T_max_) are presented as median [range]; normally distributed parameters (C_max_, AUC) are presented as mean ± SD. Normality was assessed via Shapiro–Wilk test.

Abbreviations: SD, standard deviation; T/R, Trial formulation/Reference formulation; t_1/2_, Elimination half-life; T_max_, Time to maximum concentration; C_max_, Maximum plasma concentration; AUC, area under the curve; CI, confidence interval.

Both the test and reference formulations exhibited nearly identical absorption and elimination profiles, with their plasma concentration-time curves closely overlapping, further supporting their pharmacokinetic similarities.

#### 3.2.3 Bioequivalence assessment

Bioequivalence was confirmed between the test (generic) and reference formulations, as the 90% CI for the GMR of AUC_0-24_, AUC_0-∞_, and C_max_ fell within the China NMPA regulatory acceptance range of 90.00%–111.11% (Table 6).

The C_max_ values were 18.28 ± 4.42 ng/mL for the test formulation and 18.17 ± 4.17 ng/mL for the reference formulation, with a GMR of 100.43% (90% CI: 91.57%–110.12%). The AUC_0-24_ values were 74.95 ± 21.57 ng·h/mL for the test and 75.53 ± 24.84 ng·h/mL for the reference formulation, with a GMR of 100.29% (90% CI: 94.65%–106.26%). Similarly, AUC_0-∞_ values were 76.66 ± 21.81 ng·h/mL for the test and 77.22 ± 24.97 ng·h/mL for the reference formulation, with a GMR of 100.25% (90% CI: 94.74%–106.07%).

Statistical analysis confirmed no significant differences (*P* > 0.05) between the test and reference formulations, validating their therapeutic equivalence and supporting the interchangeability of generic prazosin products. Furthermore, all subjects fell within the bioequivalence acceptance range, reinforcing the reliability and consistency of the results.

#### 3.2.4 Sex differences in pharmacokinetics

Significant sex-based pharmacokinetic variations were observed (Table 7). Although normality in some parameters, median (range) and non-parametric tests were prioritized for conservative analysis. Female subjects had a higher median C_max_ (21.80 ng/mL) compared to males (16.37 ng/mL), with a borderline P-value (*P* = 0.06). The median of AUC_0-24_ was 96.21 ng·h/mL for females and 64.81 ng·h/mL for males (*P* < 0.05), while the median of AUC_0-∞_ was 97.86 ng·h/mL for females and 66.84 ng·h/mL for males (*P* < 0.05), further confirming higher systemic exposure in females. The median of t_1/2_ was 4.02 h in females and 5.25 h in males (*P* > 0.05), suggesting comparable clearance. Likewise, the median of T_max_ was 0.75 h in females and 1.25 h in males (*P* > 0.05).

**TABLE 7:** Statistical comparison of sex differences in pharmacokinetic parameters.

Parameter	Male (n = 16)	Female (n = 4)^a^	Mann-whitney U	Exact P-value (two-tailed)
t_1/2_ (h)	5.25 [2.04–10.15]	4.02 [2.16–4.28]	21.00	0.34
T_max_ (h)	1.25 [0.50–2.50]	0.75 [0.50–2.50]	23.50	0.44
C_max_ (ng/mL)	16.37 [11.88–27.39]	21.80 [17.57–27.03]	12.00	0.06
AUC_0-24_ (ng·h/mL)	64.81 [51.33–119.80]	96.21 [79.51–120.00]	7.00	0.02
AUC_0-∞_ (ng·h/mL)	66.84 [52.91–122.30]	97.86 [80.10–121.30]	8.00	0.02

^a^ Small sample size for females (n = 4), results need further validation.

Data presented as median [range]. Non-parametric Mann-Whitney U test was applied due to small sample sizes (n = 4 in the female group).

Abbreviations: t_1/2_, Elimination half-life; T_max_, Time to maximum concentration; C_max_, Maximum plasma concentration; AUC, area under the curve.

## 4 Discussion

This study developed and validated a high-throughput LC-MS/MS method for the quantification of prazosin in human plasma, ensuring high sensitivity, accuracy, and compliance with the regulatory bioequivalence guidelines. Compared to previous methods, this approach demonstrated superior sensitivity (LLOQ: 0.1000 ng/mL), short analysis time (3.5 min), and robust extraction recovery (≥89.3%), making it well-suited for large-scale pharmacokinetic and regulatory studies. The use of prazosin-d8 as an isotopic internal standard significantly improved the matrix effect correction, ensuring consistent quantification across plasma samples. These enhancements have made this method a valuable tool for pharmacokinetic studies and therapeutic drug monitoring.

The bioequivalence study demonstrated therapeutic equivalence between the test and reference formulations, with 90% CI for AUC_0-24_, AUC_0-∞_, and C_max_ falling within the regulatory acceptance range (90.00%–111.11%), as required by China’s National Medical Products Administration (NMPA). The plasma concentration-time profiles of both formulations closely overlapped, confirming their comparable absorption and elimination patterns. Statistical analysis revealed no significant differences between formulations (*P* > 0.05), indicating that the generic prazosin formulation could be safely substituted for the reference product.

### 4.1 Sex-based differences in prazosin pharmacokinetics

One of the most significant findings of this study is the notable sex-based pharmacokinetic differences in prazosin absorption and systemic exposure. A trend toward higher C_max_ in females (*P* = 0.06) warrants further investigation. AUC_0-24_ and AUC_0-∞_ medians were 48% and 46% higher in females (*P* < 0.05). These results indicated greater systemic exposure in females, suggesting potential differences in drug absorption or metabolism. However, t_1/2_ and T_max_ did not differ significantly between sexes (*P* > 0.05), implying that drug clearance mechanisms remain similar across sexes.

Sex-based differences in pharmacokinetics have been reported for multiple drug classes and are often attributed to variations in gastric pH, enzyme activity, plasma protein binding, and body composition (Marshall and Beevers, 1996; Soldin et al., 2011). For prazosin, these factors may influence drug solubility, absorption rate, and hepatic metabolism, leading to observed differences in systemic exposure. Previous studies have highlighted that females generally have reduced first-pass metabolism, which may partially explain their higher prazosin bioavailability (Feldman and Barnett, 1991; Hatton et al., 2015). Additionally, sex-based differences in α_1_-adrenergic receptor sensitivity may further contribute to varying drug responses, influencing both efficacy and side effects (Munier et al., 2022).

### 4.2 Regulatory considerations: The need for sex-specific bioequivalence assessments

The findings of this study raise important regulatory considerations regarding the inclusion of sex-stratified analyses in bioequivalence assessments. Current bioequivalence guidelines focus on population-based pharmacokinetics without necessarily accounting for biological sex differences, despite the growing evidence of sex-related variations in drug disposition (Soldin et al., 2011; Hartmanshenn et al., 2016). Given the statistically significant differences in prazosin exposure between males and females, future regulatory frameworks should consider integrating sex-based bioequivalence evaluations, particularly for drugs with known sex-dependent metabolism or adverse effects.

Currently, China’s NMPA, the FDA, and the European Medicines Agency (EMA) do not mandate separate bioequivalence testing in male and female subpopulations, and our findings suggest that such considerations could optimize drug safety and efficacy. For instance, higher prazosin exposure in females may lead to increased risks of hypotension, dizziness, and sedation, highlighting the need for sex-specific dosing adjustments. Introducing sex-stratified pharmacokinetic analyses in generic drug approval processes could enhance precision medicine approaches and improve drug safety across diverse populations (Soldin et al., 2011; Hartmanshenn et al., 2016).

### 4.3 Clinical implications: Optimizing prazosin dosing strategies

The observed sex-based pharmacokinetic differences suggest that the current prazosin dosing guidelines may require adjustments to avoid excessive drug accumulation and heightened adverse effects in female patients. Given the vasodilatory effects of prazosin, higher systemic exposure in women may result in greater risks of hypotension, syncope, and orthostatic dizziness, potentially affecting medication adherence and patient safety (Huffman and Stern, 2007).

Despite similar elimination half-lives between sexes, the higher C_max_ and AUC in females suggest that lower doses may be sufficient to achieve therapeutic equivalence in male patients. These findings are particularly relevant for prazosin use in hypertension and PTSD, for which dose-dependent effects are well documented. Further clinical trials should investigate whether reduced doses in women can maintain therapeutic effectiveness while minimizing adverse effects (Gilpin and Weiner, 2017).

Clinicians should consider closer monitoring of female patients prescribed prazosin, especially in settings where dose-related side effects could affect treatment adherence. Additionally, individualized dosing strategies based on body weight, renal function, and metabolic differences could further refine prazosin therapy, ensuring its optimal efficacy and safety in real-world clinical practice (Khaw and Argo, 2019; Hudson et al., 2021).

### 4.4 Public health and generic drug policy implications

Beyond its clinical and regulatory implications, this study highlights the public health significance of sex-based pharmacokinetic evaluations in generic drug policy. Generic drugs play a crucial role in enhancing healthcare affordability and accessibility; however, the absence of sex-specific considerations in bioequivalence testing may introduce unintended disparities in drug safety and effectiveness (Soldin et al., 2011).

To address these gaps, policymakers should consider integrating sex-based pharmacokinetic research into regulatory frameworks to ensure that generic drugs provide equivalent therapeutic effects across all patients, regardless of sex. The inclusion of sex-based pharmacokinetic studies in generic drug approvals would improve real-world drug performance, reduce the risk of dose-related adverse events, and improve personalized medicine approaches (Marshall and Beevers, 1996).

Additionally, the global harmonization of bioequivalence guidelines should incorporate sex-stratified pharmacokinetic assessments to improve regulatory consistency and drug safety across diverse populations. Future research should focus on larger placebo-controlled trials examining sex-based differences in drug metabolism, safety, and therapeutic outcomes, ultimately guiding policy refinements in generic drug approval and clinical prescribing standards (Munier et al., 2022).

## 5 Study limitations

Despite the robustness of our validated LC-MS/MS method and the rigor of our bioequivalence assessment, this study has several limitations. First, the sample size was relatively small, particularly for female participants (n = 4), which may limit the generalizability of our findings to sex-based pharmacokinetic differences. While the observed differences in AUC support potential sex-related pharmacokinetic variability, confirmation in larger, sex-balanced studies is essential. Future trials should adopt equal enrollment strategies to investigate hormonal and metabolic influences on prazosin exposure. Second, although our study followed a single-dose, fasting-state design according to regulatory bioequivalence guidelines, it did not assess prazosin pharmacokinetics under fed conditions. Since food intake can alter drug absorption and metabolism, future studies should investigate whether similar sex-based pharmacokinetic differences persist under fed-state conditions. Third, inter-individual variability in drug metabolism was not comprehensively assessed beyond sex differences. Factors such as genetic polymorphisms in drug-metabolizing enzymes (e.g., CYP450 isoforms) and differences in renal clearance may also contribute to the observed pharmacokinetic variability. Incorporating pharmacogenetic analyses in future studies could enhance precision dosing recommendations. Lastly, while our study focused on pharmacokinetic comparability between the test and reference formulations, we did not evaluate the pharmacodynamic outcomes or adverse event profiles. Given the observed differences in drug exposure between males and females, future research should explore whether these differences translate into variations in therapeutic efficacy and safety, particularly in antihypertensive and neuropsychiatric applications of prazosin. These limitations highlight the need for further research to refine bioequivalence assessment methods, optimize individualized dosing strategies, and ensure that regulatory policies account for sex-based pharmacokinetic variability.

## 6 Conclusion

This study validated a sensitive and high-throughput LC-MS/MS method for evaluating prazosin bioequivalence, supporting regulatory compliance and clinical application. Bioequivalence between the test and reference formulations was confirmed, ensuring therapeutic interchangeability. However, significant sex-based pharmacokinetic differences were observed, with female participants exhibiting higher systemic drug exposure than male participants. These findings highlight the need for sex-stratified pharmacokinetic evaluations, support for precision medicine approaches, and regulatory policy updates. Further research is needed to explore sex-based dose adjustments to ensure optimal drug efficacy and safety. By integrating sex considerations into bioequivalence guidelines, this study contributes to advancing personalized medicine, regulatory science, and public health policies.

## Data Availability

The original contributions presented in the study are included in the article/supplementary material, further inquiries can be directed to the corresponding authors.

## References

[B1] Andros E. Detmar-Hanna D. Suteparuk S. Gal J. Gerber J. G. (1996). The effect of aging on the pharmacokinetics and pharmacodynamics of prazosin. Eur. J. Clin. Pharmacol. 50, 41–46. 10.1007/s002280050067 8739810

[B2] Chau N. P. Flouvat B. L. Le Roux E. Safar M. E. (1980). Prazosin kinetics in essential hypertension. Clin. Pharmacol. Ther. 28, 6–11. 10.1038/clpt.1980.123 7389255

[B3] Eichelman B. Dorava A. (2021). Open outcome study of prazosin in a prison female population with traumatic dreams. J. Correct. Health Care 27, 238–244. 10.1089/jchc.19.07.0062 34374567

[B4] Erve J. C. L. Vashishtha S. C. Ojewoye O. Adedoyin A. Espina R. Demaio W. (2008). Metabolism of prazosin in rat and characterization of metabolites in plasma, urine, faeces, brain and bile using liquid chromatography/mass spectrometry (LC/MS). Xenobiotica 38, 540–558. 10.1080/00498250802001826 18421626

[B5] Feldman M. Barnett C. (1991). Fasting gastric pH and its relationship to true hypochlorhydria in humans. Dig. Dis. Sci. 36, 866–869. 10.1007/BF01297133 2070698

[B6] Ferrafiat V. Soleimani M. Chaumette B. Martinez A. Guilé J.-M. Keeshin B. (2020). Use of prazosin for pediatric post-traumatic stress disorder with nightmares and/or sleep disorder: case series of 18 patients prospectively assessed. Front. Psychiatry 11, 724. 10.3389/fpsyt.2020.00724 32774309 PMC7388897

[B7] Gilpin N. W. Weiner J. L. (2017). Neurobiology of comorbid post-traumatic stress disorder and alcohol-use disorder. Genes Brain Behav. 16, 15–43. 10.1111/gbb.12349 27749004 PMC5477640

[B8] Guo X. Wu H. Guo S. Shi Y. Du J. Zhu P. (2016). Highly sensitive fluorescence methods for the determination of alfuzosin, doxazosin, terazosin and prazosin in pharmaceutical formulations, plasma and urine. Anal. Sci. 32, 763–768. 10.2116/analsci.32.763 27396658

[B9] Gurule S. Khuroo A. Monif T. Goswami D. Saha A. (2010). Rational design for variability minimization in bioanalytical method validation: illustration with LC-MS/MS assay method for terbinafine estimation in human plasma. Biomed. Chromatogr. 24, 1168–1178. 10.1002/bmc.1423 20954207

[B10] Hartmanshenn C. Scherholz M. Androulakis I. P. (2016). Physiologically-based pharmacokinetic models: approaches for enabling personalized medicine. J. Pharmacokinet. Pharmacodyn. 43, 481–504. 10.1007/s10928-016-9492-y 27647273 PMC5204363

[B11] Hatton G. B. Yadav V. Basit A. W. Merchant H. A. (2015). Animal farm: considerations in animal gastrointestinal physiology and relevance to drug delivery in humans. J. Pharm. Sci. 104, 2747–2776. 10.1002/jps.24365 25712759

[B12] Hendrickson R. C. Raskind M. A. (2016). Noradrenergic dysregulation in the pathophysiology of PTSD. Exp. Neurol. 284, 181–195. 10.1016/j.expneurol.2016.05.014 27222130

[B13] Hudson N. Burghart S. Reynoldson J. Grauer D. (2021). Evaluation of low dose prazosin for PTSD-associated nightmares in children and adolescents. Ment. Health Clin. 11, 45–49. 10.9740/mhc.2021.03.045 33850681 PMC8019540

[B14] Huffman J. C. Stern T. A. (2007). Neuropsychiatric consequences of cardiovascular medications. Dialogues Clin. Neurosci. 9, 29–45. 10.31887/DCNS.2007.9.1/jchuffman 17506224 PMC3181843

[B15] Karaoglan M. Grace A. A. (2025). A potential candidate for prevention of PTSD: prazosin prevents learned helplessness behavior in adult male rats. Psychiatry Res. 343, 116283. 10.1016/j.psychres.2024.116283 39602854 PMC12412327

[B16] Khaw C. Argo T. (2019). Prazosin initiation and dose titration in a patient with posttraumatic stress disorder on concurrent carvedilol. Ment. Health Clin. 9, 326–330. 10.9740/mhc.2019.09.326 31534876 PMC6728122

[B17] Kleinman R. A. Ostacher M. J. (2019). Prazosin and alcohol use disorder. Am. J. Psychiatry 176, 165. 10.1176/appi.ajp.2018.18101143 30704280

[B18] Liu X. Jiang J. Jin X. Liu Y. Xu C. Zhang J. (2021). Simultaneous determination of YZG-331 and its metabolites in monkey blood by liquid chromatography-tandem mass spectrometry. J. Pharm. Biomed. Anal. 193, 113720. 10.1016/j.jpba.2020.113720 33190084

[B19] Loh G. O. K. Wong E. Y. L. Tan Y. T. F. Wee H. C. Ng R. S. Syed H. K. (2022). Simple and high sample throughput LC/ESI-MS/MS method for bioequivalence study of prazosin, a drug with risk of orthostatic hypotension. Drug Dev. Ind. Pharm. 48, 470–479. 10.1080/03639045.2022.2125985 36111737

[B20] Maekawa M. Mano N. (2022). Cutting-edge LC-MS/MS applications in clinical mass spectrometry: focusing on analysis of drugs and metabolites. Biomed. Chromatogr. 36, e5347. 10.1002/bmc.5347 35073598

[B21] Marshall H. J. Beevers D. G. (1996). Alpha-adrenoceptor blocking drugs and female urinary incontinence: prevalence and reversibility. Br. J. Clin. Pharmacol. 42, 507–509. 10.1046/j.1365-2125.1996.45217.x 8904625 PMC2042702

[B22] Munier J. J. Marty V. N. Spigelman I. (2022). Sex differences in α-adrenergic receptor function contribute to impaired hypothalamic metaplasticity following chronic intermittent ethanol exposure. Alcohol Clin. Exp. Res. 46, 1384–1396. 10.1111/acer.14900 35791038 PMC9612407

[B23] National Medical Products Administration (2016). Technical guidelines for bioequivalence studies of chemical generic drugs using pharmacokinetic parameters as endpoints. Available online at: https://www.cde.org.cn.

[B24] National Medical Products Administration (2020). “Guidelines for bioavailability and bioequivalence studies of drug preparations in humans,” in Pharmacopoeia of the people’s Republic of China. 4th ed., Vol. IV (Beijing: China Medical Science Press). Available online at: https://ydz.chp.org.cn.

[B25] Rathinavelu A. Malave A. (1995). High-performance liquid chromatography using electrochemical detection for the determination of prazosin in biological samples. J. Chromatogr. B Biomed. Appl. 670, 177–182. 10.1016/0378-4347(95)00139-a 7493078

[B26] Reist C. Streja E. Tang C. C. Shapiro B. Mintz J. Hollifield M. (2021). Prazosin for treatment of post-traumatic stress disorder: a systematic review and meta-analysis. CNS Spectr. 26, 338–344. 10.1017/S1092852920001121 32362287

[B27] Seger C. Salzmann L. (2020). After another decade: LC-MS/MS became routine in clinical diagnostics. Clin. Biochem. 82, 2–11. 10.1016/j.clinbiochem.2020.03.004 32188572

[B28] Soldin O. P. Chung S. H. Mattison D. R. (2011). Sex differences in drug disposition. J. Biomed. Biotechnol. 2011, 187103. 10.1155/2011/187103 21403873 PMC3051160

[B29] Stanaszek W. F. Kellerman D. Brogden R. N. Romankiewicz J. A. (1983). Prazosin update. A review of its pharmacological properties and therapeutic use in hypertension and congestive heart failure. Drugs 25, 339–384. 10.2165/00003495-198325040-00002 6303744

[B30] The central People’s government of the People’s Republic of China. (2025).Order of the state administration of market supervision and administration (no.27).

[B31] Twomey T. M. Hobbs D. C. (1978). Analysis of prazosin in plasma by a sensitive high-performance liquid chromatographic-fluorescence method. J. Pharm. Sci. 67, 1468–1469. 10.1002/jps.2600671040 702306

[B32] Yee Y. G. Rubin P. C. Meffin P. (1979). Prazosin determination by high-pressure liquid chromatography using fluorescence detection. J. Chromatogr. 172, 313–318. 10.1016/s0021-9673(00)90967-1 548534

